# Why Evidence Is Not Enough: Power, Politics, and a Strategy Shift for Public Health

**DOI:** 10.1111/1468-0009.70116

**Published:** 2026-08-08

**Authors:** JONATHAN C. HELLER

**Affiliations:** ^1^ Population Health Institute University of Wisconsin & the Health & Power Organizing Project

**Keywords:** health equity, structural determinants of health, power, community organizing, narrative infrastructure

## Abstract

**Policy Points**

- Public health has lost political influence because of a mismatch between the forms of power primarily deployed in this field—knowledge and moral authority—and the forms of power that currently shape societal rules and health outcomes—economic, political, ideological, and physical.
- Rebuilding the influence of public health requires a strategy shift that returns the field to its roots: building people power through partnerships with labor and community organizing groups; strengthening ideological power through the development of narrative infrastructure; and in moments of institutional breakdown, deploying nonviolent disruptive power to protect population health.

**Context:**

Public health is inherently political, yet it has struggled in recent decades to influence the societal decisions that shape population health. Despite strong evidence supporting policies across domains such as environmental protection, infectious disease control, labor conditions, and reproductive health, the field has faced growing political backlash and policy retrenchment. This Perspective argues that these challenges reflect a mismatch between the forms of power public health primarily deploys and the forms of power that currently shape societal rules and health outcomes.

**Analytic Approach:**

This Perspective draws on an established typology of power to conceptually analyze the forms of power available to public health practitioners and to actors advancing policies and institutional arrangements detrimental to public health.

**Findings:**

Public health has relied primarily on knowledge and moral power, whereas actors advancing policies detrimental to health increasingly wield economic, political, ideological, and physical power within a neoliberal political economy. Under conditions of polarization, disinformation, and institutional capture, knowledge and moral power alone are structurally insufficient to shape the societal rules that determine health. The analysis identifies additional forms of power available to public health—including people, ideological, and nonviolent disruptive power—that could strengthen the field's political influence while remaining consistent with health equity values.

**Conclusions:**

The analysis suggests a strategic shift for public health: rebuilding people power through partnerships with labor and community organizing groups, strengthening ideological power through sustained investment in narrative infrastructure, and, when democratic institutions fail to protect health, joining with others in the strategic use of nonviolent disruptive power. More broadly, advancing population health will require public health to engage more deliberately with power and politics rather than relying on evidence alone.

By many measures, including life expectancy, substance use mortality, infectious disease control, and environmental protection, public health in the United States has lost influence over the decisions that shape population health. Although the field achieved major gains in the 20th century, including reducing tobacco use, improving workplace and motor vehicle safety, and expanding vaccination, public health practitioners have struggled in recent decades to secure comparable advances, particularly in reducing inequities. The COVID‐19 vaccine rollout demonstrated public health's scientific and operational capacity, yet measures such as masking and vaccine mandates led to a backlash that weakened public health authority and constrained the ability of public health practitioners to act.

Public health has long faced opposition, from resistance to gun violence research to reproductive health and harm reduction policies. However, these conflicts have intensified in today's highly polarized political environment, exposing the field's limited ability to shape the societal rules that determine health. This Perspective argues that the political marginalization of public health—and more specifically of health‐equity–focused public health practitioners, researchers, educators, and funders working within government, nonprofits, academia, and foundations who are the focus of this Perspective—reflects not only an imbalance of power, but a mismatch between the forms of power public health primarily deploys and the forms of power that now govern societal decision making.

Understanding this mismatch requires an analysis of power dynamics. What forms of power has public health wielded historically and in recent decades? What forms of power have those advancing worldviews, governance structures, laws, policies, and institutional practices that are detrimental to public health been able to wield? What forms of power could public health develop to more effectively shape the conditions that determine health? Only by answering such questions will it be possible to develop strategies capable of advancing health and equity.

Although public health encompasses a range of perspectives and approaches, the field of public health, that is, the institutions, professionals, and knowledge systems focused on shaping the conditions that produce health, is inherently political.^1^, ^2^ Achieving improvements in health requires shaping the structural determinants of health: the written and unwritten rules, along with the power dynamics behind them, that create and preserve durable and hierarchical patterns of advantage among socially constructed groups in the conditions that affect health.^3^ These rules include values, beliefs, worldviews, cultures, and norms; governance; laws, policies, regulations, and budgets; and institutional practices.^3^ Those who benefit from unjustly marginalizing and profiting off of others have disproportionate power to influence societal rules to advance their own interests. Many of the rules that they have been able to enact have the consequence, intended or not, of directly or indirectly harming the health of those with less power as a result of social hierarchies based on class, race, immigration status, gender identity, sexual preference, and other dimensions of identity; these rules are therefore incompatible with public health goals. Ultimately, the distribution of power to shape societal rules plays a central role in influencing which populations are more or less protected in their health.

## Power Framework

Individual and community power have been linked to health and health equity outcomes through both theoretical and empirical research.^4^, ^5^ Although the literature contains many definitions of power, Dr. Martin Luther King Jr defined it simply as “the ability to achieve a purpose.”^6^ ^(p199)^

There are many frameworks for conceptualizing and analyzing power, several of which have been applied in public health.^7^ One well‐established approach to analyzing power that can offer insight into public health strategy examines how actors derive power from specific sources.^8^ Drawing from this approach, I focus on eight of these sources, or forms, of power, namely, knowledge, economic, political, ideological, physical, people, and disruptive, as described in Table 1.^8^, ^9^, ^10^, ^11^ ^(pp44‐57)^ Use of this typology allows for the identification of the types of power currently used by health‐equity–focused public health practitioners, researchers, educators, and funders, along with the types of power systematically underutilized relative to other actors shaping the public's health. There are other forms of power described in the literature that are not considered here because they are currently less relevant for public health, although they could be future sources of public health power.

**Table 1:** Forms of Power

Form of Power	Source From Which Power Is Derived
Knowledge	Information and technical expertise
Moral	Shaping the principles and actions that others believe to be right or wrong
Economic	Material resources, primarily through controlling the production of goods and services and through wealth
Political (or state)	Governing structures that legitimize the ability to regulate, punish, discipline, and (re)distribute resources
Ideological (or discursive or narrative)	Shaping how people make sense of the world and understand issues and events
Physical (or military)	Use or threat of force or violence
People (or solidarity)	People in relationship, working together in networks and alliances
Disruptive	Withholding consent, resisting, or noncooperation with the status quo

Data derived from Moon^10^ and Bhargava and Luce.^11^

## Public Health's Primary Sources of Power

### Knowledge Power

Whereas public health's roots include close allyship with people‐powered social movements, the field largely shifted away from that approach a century ago in favor of knowledge power.^12^ Public health today emphasizes its objectivity and evidence‐based practice.^13^, ^14^ A common implicit theory of change in public health is that, through education, the public will understand and act on information about health and policymakers will implement evidence‐based policies that advance public health goals. Many approaches to public health have operated under assumptions embedded in this theory of change: that individual behavior change is enough to improve health and that behavior change can overcome structural barriers to health.

Amid threats to public health, the field falls back on evidence in its communication, litigation, and administrative responses. For example, the American Public Health Association's communications responding to the recent overhaul of vaccine recommendations by the Centers for Disease Control and Prevention's Advisory Committee on Immunization Practices called for an evidence‐based recommendations process, provided statistics, and emphasized that links between vaccines and autism had been debunked.^15^, ^16^

Knowledge power has historically been effective when political consensus aligned with public health goals, for example, the Biden administration's Executive Orders requiring masking and social distancing on federal properties. However, knowledge power is likely to be structurally insufficient in contexts where it is in tension with entrenched economic, political, or ideological interests. Reliance on knowledge power has neither protected public health nor advanced evidence‐based solutions, with recent immunization policy changes being a clear example.

When communications and advocacy present data that conflict with people's worldviews, those individuals dismiss the findings.^17^ When data threaten monied interests, on issues including tobacco and climate change, for example, those interests may counter and suppress the data or fund countervailing research. In practice, this suggests that ideological power and economic power often override knowledge power and that policymakers instead engage in “policy‐based evidence making.”^18^

Critics of neoliberal capitalism argue that appeals to knowledge resonate most with the “professional class”—a group that includes public health professionals—who seek to separate science from politics, but less so with the working class, who may not feel that the evidence presented matters in their lives.^19^ ^(pp109‐142)^ Emphasizing a separation between science and politics obscures the reality that decisions about whether and how evidence is acted upon are inherently political and shaped by other forms of power.

Those whose priorities do not align with public health and/or health equity goals, such as fossil fuel and tobacco companies, have challenged or sought to undermine what the field of public health considers to be its greatest strength, by weakening knowledge power through disinformation. Social media platforms, driven by profits, have amplified the spread of misleading information.^20^ These dynamics are evident across many contemporary public health issues, including COVID‐19 safety measures and vaccine policy.

Public health uses other forms of power, including those discussed in the “Forms of Power Used by Those Advancing Societal Rules Detrimental to Public Health” and “Moving Forward: Building Additional Forms of Power for Public Health” sections, but it has relied on them less, wielded them less effectively, and/or has less of them than actors advancing agendas harmful to health. This analysis does not suggest abandoning data and evidence, but rather strengthening knowledge power and using it along with other forms of power. Strengthening knowledge power requires epistemic justice, expanding what counts as valid knowledge and whose knowledge is recognized as valid by, for example, valuing lived experience as evidence; incorporating qualitative data as causal insight; collecting, analyzing, and reporting community‐defined indicators of health and well‐being; and interpreting evidence through historical and structural lenses.^21^, ^22^ It also requires democratizing data, including supporting community‐led data collection and interpretation, addressing data sovereignty issues, increasing data transparency, redistributing the capacity to analyze data, and coproducing knowledge between public health institutions and communities.^23^, ^24^ Doing so broadens the legitimacy of evidence, enabling more people to see their experiences reflected in it, in effect making knowledge power more connected to people power and, thereby, more influential.^25^

### Moral Power

Public health has also relied on moral persuasion, most prominently in anti‐smoking campaigns.^26^ Studies of the effectiveness and acceptability of moral appeals, however, show mixed results,^27^ and such appeals can lead to stigmatization of some populations, including, for example, people with HIV/AIDS and people who have been deemed overweight.^28^ Moral arguments have also been used by those opposed to a health equity agenda, including by anti‐abortion activists. Moreover, it is unclear whether moral arguments currently hold substantial influence, with the such appeals often being dismissed, including on civil rights issues.^29^

Research on the civil rights movement suggests that moral arguments were important but not sufficient; social, economic, and ideological disruption were critical to achieving structural change.^30^

Again, this analysis does not suggest that public health should completely abandon moral power. Rather, public health practitioners should (1) ensure that they use moral power in ways that do not stigmatize people for their behaviors; (2) strategically incorporate moral arguments that resonate with large majorities of people in their communications; and (3) use moral power in combination with other forms of power, including people power and disruptive power, as described in the “Moving Forward: Building Additional Forms of Power for Public Health” section. For example, one might call out the immorality of extreme wealth inequality and the behavior of billionaires in creating it, align with social movements fighting for a more just allocation of society's wealth, and present public health strategies that redistribute wealth as a more principled approach.

## Forms of Power Used by Those Advancing Societal Rules Detrimental to Public Health

### Economic and Political Power

Under neoliberalism—the political and economic framework that has prevailed over the past half century, often associated with an emphasis on market‐based solutions, individual liberty, and limited government, and with upward shifts in wealth and power^31^—monied interests have gained outsized influence over the societal rules governing the US economy and political system. These rules and the economic system they combine to create—that is, the commercial determinants of health^32^—are key structural determinants of health. Through deregulation, privatization, consolidation, and related processes, many social determinants of health, including food, education, health care, work, transportation, and social connections, have been reshaped in ways that are harmful.^33^

Monied interests currently have more influence over economic systems than community‐controlled organizations and democratically elected bodies operating independently of those monied interests.^34^ Through deregulation, governmental institutions have increasingly ceded authority to corporations. Changes in campaign finance rules, including through the Supreme Court's Citizens United decision, have further expanded corporate influence over democratic governance.^35^ As a result, government institutions increasingly reflect the priorities of economic interests. For example, the American Petroleum Institute has lobbied the Environmental Protection Agency to change how it calculates the value of human life when setting air pollution limits; indeed, the Environmental Protection Agency recently effectively set the value at zero dollars, a decision with clear public health consequences.^36^

Economic power is not a source of power that public health can easily build, and the available methods of amassing the necessary wealth to exercise economic power are not aligned with a health equity agenda. Such wealth is often generated through activities that are harmful to health, including environmental degradation, creation of unsafe working conditions, and the sale of harmful products. However, public health can use other forms of power to constrain corporate economic power, for example, through environmental, worker protection, and antitrust regulation, and it can help build democratic control over the economy, including through strengthening labor unions and democratic institutions.^34^

The choice of public health to not engage politically cedes influence over the structural determinants of health to these monied interests. Public health can, instead, build values‐aligned forms of power to ensure that political and economic systems improve the public's health. Efforts to do so, including initiatives to strengthen democratic governance, are underway.^37^

### Ideological Power

Neoliberal ideology enabled the rise of corporate economic power. By glorifying individual responsibility and free markets, discrediting government intervention, and denying structural oppression, this ideology contributes to health inequities.^33^, ^38^ When widely adopted, these beliefs become embedded in institutions and norms, and they become more powerful than evidence. Neoliberal ideology was advanced through a range of institutional and policy processes,^31^ and media outlets such as Fox News—enabled by media deregulation in the 1980s—continue to reinforce it.

Public health can build ideological power through the development of a narrative infrastructure, thereby increasing its power to shift worldviews that stand in the way of advancing health equity.^39^ Although efforts at narrative change are growing,^40^ practitioners in the field of public health have learned from those advancing neoliberalism that transforming dominant ideology requires sustained funding, coordination, and long‐term commitment. It remains unclear whether public health will receive the necessary support, for example, from philanthropies, to compete ideologically or whether building ideological power alongside people power can achieve similar ends.

### Physical Power

The use and threat of police force have major public health impacts, including death, injury, trauma, and fear, with disproportionate effects on people of color. Public health can develop strategies backed by people and disruptive power, as described in the following section, to respond to physical power, to prevent such harm.

Public health has also used coercive power, backed with the threat of physical power, to advance its agenda, for example, with infectious disease control.^41^ However, because this approach does not align with a health equity agenda—coercive practices typically undermine the trust‐building with marginalized communities that must be done to advance health equity—building physical power should not be a focus of health‐equity–focused public health practitioners, researchers, educators, and funders.

## Moving Forward: Building Additional Forms of Power for Public Health

Given this analysis, I now turn to these remaining questions: *What types of power might public health be able to build moving forward? What strategies need to be employed to build those forms of power?* I consider two forms of power, namely, people power and disruptive power, to begin to lay out potential strategies.

### People Power

People power is consistent with health‐equity values and working with social movements, the manifestation of people power, was central to public health's early successes in improving housing, sanitation, and working conditions.^12^ The field largely shifted away from such work, however, starting in the 1920s, as bacteriology and laboratory science became dominant.^12^

In the face of the economic, political, ideological, and physical threats, rebuilding connections to and supporting the strengthening of people power—through worker and community organizing—is essential. The theory of change underlying organizing is that change requires collective action that shifts power relations. By organizing within public health and in partnership with labor and community groups, practitioners of public health can help shift power relations and advance healthier workplace and community conditions. Public health workers can show up as allies, build trust with organizers and communities, support their work, and strategize with them.

Building people power requires partnership with groups organizing across marginalized populations and geographies. When public health has partnered with organizing groups, including those that anchored recent movements such as Black Lives Matter and the Fight for $15, health‐equity–focused public health practitioners, researchers, educators, and funders have typically worked with those organizing the groups most marginalized by society. However, because people power is predicated on large numbers and because economically exploited white working‐class communities are a large constituency also facing significant health problems, public health should also partner more closely with these communities. Such partnership can start with dialogue and listening but must then move to the authentic deep engagement enabled by organizing.

Although such work is currently uncommon in public health, by supporting organizing around a community‐defined agenda, one that might include wages, jobs, housing, environmental conditions, education, and health care, and not simply its own priorities, public health could build trust and strengthen its impact.^42^ Building trust is possible because organizing practice deliberately focuses on forming deep, reciprocal relationships through collective struggle, and such relationships are the foundation of trust.^43^ This approach can be understood as building a popular base of support for public health or, more radically, as reimagining the field to include the wider ecosystem of groups working to improve the public's health.

As recently described, there are many ways for public health to engage with organizing groups both to build people power and to make its other forms of power, such as knowledge and ideological, more effective.^44^ Public health actors can build long‐term trusting relationships with labor and community organizing groups, develop an understanding of the problems people in those groups are facing, and act to support those groups as they work to achieve their goals. The positionality that comes with a public health role can be used to help organizers build relationships with others who support this agenda and bring the members of these organizing groups into spaces and processes to which public health practitioners have access, when they do not. Those active in the field of public health can partner with organizing groups to conduct research on issues they prioritize, work with them to collect new data, analyze data and evidence with them to inform their work, and develop reports to reframe issues using a lens focused on social and structural determinants of health. Public health can also support organizing groups as they develop their campaigns and policy agendas and conduct advocacy in support of the community's agenda. Last, public health practitioners can modify and orient their public health interventions to address the root causes of the inequities prioritized by organizing groups.

A recent rapid review of the literature on public health's work with community organizing groups found several challenges to this work.^42^ These include administrative barriers, such as the availability of funding for these partnerships and differing project timelines; approach differences, such as communication styles and public health's prioritization of objectivity; and the challenges inherent in community organizing, such as the labor‐intensive work required of organizers and the need to ensure that a community organizing group is truly representative of a community.^42^ The review found that these challenges “are operational in nature and may be overcome through efforts such as investing time in building relationships, fostering trust, or gaining skills and competence.”^42^

Advancing a “people's public health agenda” in this way can challenge the power of monied interests and transform the structural determinants of health. Although the field of public health has much to (re)learn about doing this, there are recent examples from which progress can be built, such as the Health and Power Organizing Project, which, by organizing health justice scholars, is building the collective power of and increasing the understanding of organizing among the public health community;^45^ Power‐building Partnerships for Health, which supports local health departments and community organizing groups in working together;^46^ Defend Public Health, which has started state‐based organizing for public health,^47^ and funder‐led efforts including The California Endowment's Build Healthy Communities initiative^5^ and the Robert Wood Johnson Foundation's Lead Local project.^48^

### Disruptive Power

Many leaders in public health and beyond warn that the United States is facing democratic backsliding. Undoubtedly, the tactics recent federal administrations have used, for example, for immigration enforcement, are causing health harms to structurally marginalized communities. When formal governance systems are insufficient to protect health, disruption becomes a necessary tool for harm prevention.

Disruptive power works by withholding consent and interrupting business as usual.^49^ It involves challenging institutional arrangements that sustain harmful systems and imposing material, political, and/or economic costs until change occurs. Strategic disruption targets potential system vulnerabilities for a sustained period and uses tactics that clarify moral stakes while creating pressure, as seen in civil rights boycotts and sit‐ins.^11^ ^(p54)^

Disruptive power involves nonviolent tactics, including censorship noncompliance, sit‐ins, boycotts, and public art. It can include interrupting key public health meetings and providing health care when it has been made illegal. Effective disruption can also include providing mutual aid. Although these actions carry risk, by expressing joy and love they can also be radical acts of resistance.

Public Health can learn from movements such as the AIDS Coalition to Unleash Power (more commonly known as ACT‐UP), whose nonviolent disruption, involving blocking streets, interrupting newscasts, and mobilizing thousands of people to shut down the Food and Drug Administration, transformed the response to the AIDS crisis.^50^ In this case, public health was the target of the use of disruptive power by a marginalized community, and this disruption ultimately advanced health equity. Those working in the field of public health must learn to resist the temptation to push back against such outside disruption, for example, because of a disagreement with the tactics used and, instead, embrace it as creating political space for the field to more aggressively advance health equity and channel it toward necessary institutional change. Response to outside disruption requires nuanced and thoughtful analysis, as such disruption can also come from forces opposed to health equity, and in these cases, health equity advocates must use other forms of power to push back. It is necessary to develop an analysis of the underlying interests of those targeting public health using disruptive power and respond accordingly.

Building public health's disruptive power, and responding appropriately in the face of disruptive power, will require creativity, coordination, and courage to defend public health and health equity and to prevent harmful policies.

## Conclusions

Answering the questions posed at the start of this Perspective has made clear the power imbalance and mismatch faced by public health. Over the past century, public health, including health‐equity–focused public health practitioners, researchers, educators, and funders, have relied primarily on knowledge power and, to a lesser extent, moral power. In contrast, over the past few decades, those advancing societal rules harmful to health have wielded economic power, built ideological power, captured political institutions, and used physical power to maintain the status quo. Moving forward, public health could return to its roots, aligning with those building people power and, when necessary, using disruptive power.

This analysis suggests five strategic shifts for public health: First, build *people power* by forming deep, trusting relationships with labor and community organizing groups and supporting a people's public health agenda focused on the determinants of health that are of shared interest. Second, develop *ideological power* through sustained investment in narrative infrastructure. Third, strengthen *knowledge power* by democratizing data and evidence and using them alongside other forms of power. Fourth, join with others in using *disruptive power* to prevent harm. Fifth, use these forms of power to disrupt the *economic and political power* of monied interests and to strengthen democratic control over economic and political systems.

This strategy is unlikely to be implemented by current public health leaders within existing public health structures. Health‐equity–focused practitioners, researchers, educators, and funders will need to organize, develop new leaders and structures, and build their power to shape the field as it re‐emerges and recreates itself in the future.

Advancing public health and health equity require engaging directly with power and politics. Without doing so, public health risks further marginalization, continued distortion of health equity initiatives, and additional harm to the communities served. Public health faces a crucial strategic decision: rely on evidence alone and hope those in power act, or realign the field's strategies to build the power needed to reshape the rules that determine population health.

## Funding/Support

This work was supported in part by grant funding from the Freedom Together Foundation and the Robert Wood Johnson Foundation. The views expressed herein do not necessarily reflect the views of the funders or the University of Wisconsin Population Health Institute.

## Conflict of Interest Disclosures

No conflicts of interest were reported.
